# Long-term protective effect of high-risk human papillomavirus testing in population-based cervical screening

**DOI:** 10.1038/sj.bjc.6602541

**Published:** 2005-04-12

**Authors:** N W J Bulkmans, L Rozendaal, F J Voorhorst, P J F Snijders, C J L M Meijer

**Affiliations:** 1 Department of Pathology, VU University Medical Center, PO Box 7057, 1007 MB Amsterdam, The Netherlands; 2 Department of Clinical Epidemiology and Biostatistics, VU University Medical Center, Amsterdam, The Netherlands

**Keywords:** human papillomavirus, cervical cancer, screening, long-term

## Abstract

We prospectively evaluated the 5-year predictive values of adding high-risk human papillomavirus (hrHPV) testing to cytology for the detection of ⩾cervical intraepithelial neoplasia (CIN)3 lesions in a population-based cohort of 2810 women. At baseline, nine (0.3%) women had prevalent lesions ⩾CIN3, all being hrHPV positive. After 5 years of follow-up, four (6.5%) of the 62 hrHPV-positive women with normal cytology developed lesions ⩾CIN3, *vs* only one (0.05%) of the 2175 hrHPV-negative women with normal cytology. High-risk human papillomavirus testing or combined screening revealed a much higher sensitivity, at the cost of a small decrease in specificity, and a higher negative predictive value for the detection of lesions ⩾CIN3 till the next screening round (5 years) than cytology alone.

Cervical screening by cytology is known to yield a substantial proportion of both false-positive and false-negative smears. Since infection with high-risk human papillomavirus (hrHPV) is considered to be the cause of cervical carcinoma, it has been suggested that adding hrHPV testing to cervical screening might improve screening in terms of reducing false-positive and false-negative smears (Rozendaal *et al*, 2000; Cuzick *et al*, 2003). However, long-term data are needed before hrHPV testing can be implemented in population-based cervical screening. Here, we present for the first time the prospective long-term (5 years) predictive values of routine hrHPV testing in population-based cervical screening in The Netherlands.

## MATERIAL AND METHODS

A cohort of 3170 women (mean age 45 years; range 29–61 years) was enrolled from March 1995 till October 1998 by 60 general practitioners in the small district of Amstelveen, The Netherlands. In all, 194 women were excluded because of abnormal cervical cytology and/or histology during 2 years preceding the intake, 159 women because of a negative *β*-globin PCR test, and seven women because of inadequate cytology, leaving 2810 women for analysis. Cytological screening was performed according to the CISOE-A classification, routinely used in cervical screening in The Netherlands, and the referral policy was according to the nationwide guidelines (Bulk *et al*, 2004). High-risk human papillomavirus testing was performed by GP5+/6+ PCR-EIA, using a cocktail probe of 14 hrHPV types, and independent of cytology results. The hrHPV test result was blinded. Informed consent was obtained and the study was approved by the Medical Ethics Committee of the VU University Medical Center. The primary end point of the study was the detection of histologically proven cervical intraepithelial neoplasia grade 3 or cervical carcinoma (⩾CIN3) up to and including the next screening round (after 5 years). Follow-up data were retrieved from the Dutch nationwide pathology registry (PALGA) in 2004.

Sensitivity, specificity, negative predictive value (NPV) and positive predictive value (PPV) of cytology, cytology and hrHPV testing combined, and hrHPV testing, for the detection of prevalent and incident lesions ⩾CIN3 were computed using two by two tables. Results are presented as percentages with 95% confidence intervals (95% CI). To determine whether any gain in test performance from the addition of the second test (hrHPV testing) was greater than that expected if the second test offered no diagnostic information, expected performance characteristics for cytology combined with a random test having the similar prevalence as hrHPV were computed (Franco and Ferenczy, 1999).

## RESULTS

Of 2810 women for analysis at baseline, 2687 (95.6%) women had normal cytology, of whom 77 (2.9%) had a positive hrHPV test. Among 111 (4.0%) women with borderline or mild dyskaryosis (BMD), 16 (14.4%) women were hrHPV positive, as were 11 (91.7%) of 12 (0.4%) women with moderate dyskaryosis or worse (>BMD). Among the 123 (5.2%) women who had abnormal cytology, nine (7.3%) cases of prevalent lesions ⩾CIN3 were present (including three squamous cell carcinomas) (Table 1), resulting in a detection rate of 0.3% lesions ⩾CIN3 by cytology among all 2810 women at baseline. All women with lesions ⩾CIN3 at baseline had a positive hrHPV test.

The median follow-up time until histological or cytological diagnoses was 4.6 years (range 0.1–8 years). The follow-up results of women with normal cytology at baseline are presented in Table 2. Of the 2687 women with normal cytology, 304 did not have follow-up data registered in PALGA. Another 146 women were excluded because they reached age 60 years or over and therefore were not called for further screening, leaving 2237 (83.3%) women with normal cytology for follow-up analysis. Among the 62 women of this cohort with an hrHPV-positive test at baseline, four (6.5%) cases had incident lesions ⩾CIN3. Among the 2175 women with a negative hrHPV test, one (0.05%) lesion ⩾CIN3 (an adenocarcinoma) was registered within this follow-up period. This woman with a symptomatic adenocarcinoma, who was tested hrHPV negative by GP5+/6+ PCR EIA twice, revealed human papillomavirus (HPV) type 31 by E7 type-specific PCR in the baseline and follow-up smear. This suggests viral integration with disruption of the L1 region that is targeted by the GP5+ and GP6+ primers. For hrHPV-positive women with normal cytology, the relative risk to develop lesions ⩾CIN3 in 5 years was 140.3 (95% CI; 15.9–1237.3) compared to hrHPV-negative women with normal cytology. The overall detection rate after 5 years in this study of lesions ⩾CIN3 by cytology was 0.5%.

The performance characteristics of cytology, cytology and hrHPV combined, and hrHPV testing, for the detection of lesions ⩾CIN3 are given in Table 3. Combined screening revealed a much higher sensitivity, at the cost of a small decrease of specificity, and a higher NPV at the next screening interval (after 5 years) than cytology alone. Combined testing was more sensitive than cytology alone (92.9 *vs* 64.3%, respectively; *P*=0.065), but was less specific (92.7 *vs* 95.14%, respectively; *P*<0.001), and had an increased NPV (99.95 *vs* 99.78%, respectively; *P*=0.109) with a similar PPV (7.0 *vs* 7.3%; *P*=0.922). High-risk human papillomavirus testing alone was more sensitive than cytology alone (92.9 *vs* 64.3%, respectively; *P*=0.065), more specific (96.8 *vs* 95.1%, respectively; *P*=0.005), and had an increased NPV (99.96 *vs* 99.78%, respectively; *P*=0.098) and an increased PPV (14.6 *vs* 7.3%, respectively; *P*=0.085). Comparing the sensitivity and NPV of cytology and hrHPV combined screening to cytology with a random test, no different significance levels were revealed as when compared to cytology alone.

## DISCUSSION

Combined screening revealed a much higher sensitivity, at the cost of a small decrease of specificity, and a higher NPV at the next screening interval (after 5 years) than cytology alone. Cytology alone had reasonable performance characteristics, but on adding hrHPV testing we can achieve much better performance characteristics in population-based screening.

The overall detection rate by cytology of 0.3% lesions ⩾CIN3 at baseline and 0.5% after 5 years in this study is comparable to the detection rate of lesions ⩾CIN3 in cervical cancer screening in The Netherlands (Bos *et al*, 2002; Anttila *et al*, 2004). The high NPV of the combination of a negative hrHPV test and a normal smear is in accordance with Clavel *et al* (2004), who reported an NPV of 99.9% in a partly hospital-based population, with a much shorter interval (median 2.8 years) for women of 15–79 years of age. Sherman reported an NPV of 99.2% for hrHPV in cervical lavage specimens during annual screening for women of 16–94 years of age (Sherman *et al*, 2003). The data are also in line with the data of the Manchester cohort (Peto *et al*, 2004).

Our data are the first that were prospectively obtained in population-based screening, with a 5-years screening interval in women 30–60 years of age. Some methodological aspects of this study need to be discussed. The fact that histology was not obtained in all women might induce a verification bias in advantage of combined screening and hrHPV testing. However, women were followed according to current practice standards in nationwide screening with cytology after 5 years, and in this setting women with normal cytology are considered to be free of disease. As indicated by the overlapping 95% CIs, the gains in sensitivity and NPV by combined testing or hrHPV testing compared to cytology alone were not significant (*P*⩽0.05). This might be due to our relatively small population with a low prevalence of lesions ⩾CIN3, resulting in wide 95% CIs. However, with this low prevalence of lesions ⩾CIN3, still a borderline significance was reached (*P*⩽0.1). The increase in sensitivity by combined testing compared to cytology alone may be misleading because improvements in sensitivity would be expected by adding a second test, even if the second test performed randomly with respect to disease identification. For this reason, expected performance characteristics for cytology combined with a random test were computed to determine if any gain in test performance from the addition of the second test (hrHPV testing) was greater than that expected if the second test offered no diagnostic information (Franco and Ferenczy, 1999). By comparing the sensitivity and NPV of cytology and hrHPV combined screening to cytology with a random test, no different significance levels were revealed as when compared to cytology alone.

Cost-effectiveness studies and modelling studies show that cervical screening may become much more efficient in terms of decreasing numbers of false-negative and false-positive smears, if a test is used with a substantial higher sensitivity and long-term NPV than conventional cytology (Canfell *et al*, 2004). Negative test results in combined screening predicted that the future risk for lesions ⩾CIN3 was very low. The higher sensitivity for lesions ⩾CIN3 and the long-term NPV for lesions ⩾CIN3 of hrHPV testing in combination with classical cytology and of sole hrHPV testing show that these could be such a test.

The use of hrHPV testing in cervical screening could lead to several different screening strategies, including combined cytology and hrHPV testing, or primary screening by hrHPV with cytology reading only of women tested hrHPV positive. In addition, the high sensitivity and NPV of hrHPV testing opens possibilities for longer screening intervals with still acceptable rates of incipient lesions. Modelling studies and confirmation of our results in larger studies will help to clarify this discussion and to devise more efficient cervical screening strategies.

## Figures and Tables

**Table 1:** Baseline histology stratified to cytological diagnosis and hrHPV status

		**Scc**	**Aden. Ca**	**CIN3**	**CIN2**	**Lesser abnormality**	**Normal**	**Total**
**Baseline cytology**	**Baseline hrHPV**	***n* (%)**	***n* (%)**	***n* (%)**	***n* (%)**	***n* (%)**	***n* (%)**	***n* (%)**
Normal	**+**	—	−	—	—	—	77 (100)	77 (100)
	−	—	−	—	—	—	2610 (100)	2610 (100)
BMD	**+**	—	−	3 (18.8)	3 (18.8)	2 (12.5)	8 (50.0)	16 (100)
	−	—	−	—	1 (1.1)	6 (6.3)	88 (92.6)	95 (100)
>BMD	**+**	3 (27.3)	−	3 (27.3)	2 (18.2)	3 (27.3)	—	11 (100)
	−	—	−	—	—	—	1 (100)	1 (100)
Total		3 (0.1)	−	6 (0.2)	6 (0.2)	11 (0.4)	2784 (99.1)	2810 (100)

Scc, squamous cell carcinoma; Aden. Ca, adenocarcinoma; CIN1–3, cervical intra-epithelial neoplasia grade 1–3; lesser abnormality, CIN1 or abnormal smear; normal, histological or cytological normal diagnoses; BMD, borderline or mild dyskaryosis (Pap 2–3a mild dyskaryosis); >BMD, moderate dyskaryosis or worse (Pap 3a moderate dyskaryosis or worse); FU, follow-up. Baseline histology is presented for women with BMD and >BMD, with the annotation that for women with BMD histology was obtained after a serial abnormal smear after 6 or 18 months. For women with normal cytology (Pap 1), the follow-up data are presented in Table 2.

**Table 2:** Years of follow-up of women with normal cytology stratified to final histological diagnosis

		**0 year**	**1 year**	**2 years**	**3 years**	**4 years**	**5 years**	**6 years**	**7 years**	**8 years**	**Total FU**
**Baseline status**	**FU**	***n***	***n***	***n***	***n***	***n***	***n***	***n***	***n***	***n***	***n***
Normal cytology, hrHPV positive	⩾CIN3	—	1	—	—	2^a^	1	—	—	—	4
	CIN2	—	—	—	—	—	—	—	—	—	—
	Lesser abnormality	—	—	1	—	2	3	—	—	—	6
	Normal	—	4	7	6	10	19	4	1	1	52

Normal cytology, hrHPV negative	⩾CIN3	—	—	—	—	—	—	—	1^b^	—	1
	CIN2	—	—	2	1	—	—	—	—	—	3
	Lesser abnormality	1	—	—	3	2	13	6	1		26
	Normal	19	60	252	338	337	723	346	60	10	2145

Total		20	65	262	348	353	759	356	63	11	2237

Of the total number of 2687 women with normal cytology, 450 did not have follow-up data: 146 women of 59–61 years of age were not expected to have another cervical screening and 304 did not have another screening because of other reasons. *FU*, follow-up; *year(s)*, year(s) of follow-up; *CIN*, cervical intra-epithelial neoplasia; ⩾*CIN3*, CIN3 or worse; *lesser abnormality*, CIN1 or abnormal smear; *normal*, Histological or cytological normal diagnoses.

a One woman was diagnosed with squamous cell carcinoma, the other with CIN3.

b Adenocarcinoma.

**Table 3:** Observed performance characteristics for lesions ⩾CIN3 for cytology, cytology+hrHPV and hrHPV testing

	**Sensitivity**	**Specificity**	**Positive predictive value**	**Negative predictive value**
Cytology	64.3 (35.6–86.0)	95.1 (94.2–96.0)	7.3 (3.9–13.3)	99.78 (99.48–99.90)
Cytology+HrHPV	92.9 (64.2–99.6)**	92.7 (91.5–93.7)*	7.0 (4.2–11.7)†	99.95 (99.74–99.99)**
HrHPV	92.9 (64.2–99.6)**	96.7 (95.9–97.4)*	14.6 (8.7–23.4)**	99.96 (99.75–99.99)**

Expected performance characteristics for cytology and a combined test, which assumes that the second test is random with the same prevalence as hrHPV (3.7%), with respect to disease detection: sensitivity 65.6 (36.8–86.9), specificity 91.6 (90.3–92.6), PPV 4.4 (2.4–8.2), NPV 99.78 (99.47–99.91); *P*-values for differences in performance characteristics compared to cytology:

* *P*⩽0.05;

** *P*⩽0.1;

† *P*>0.1.
